# Effect of remote online exam delivery on student experience and performance in applied knowledge tests

**DOI:** 10.1186/s12909-021-02521-1

**Published:** 2021-02-02

**Authors:** Alan Jaap, Avril Dewar, Colin Duncan, Karen Fairhurst, David Hope, David Kluth

**Affiliations:** Medical Education, Edinburgh Medical School, University of Edinburgh, Edinburgh, UK

**Keywords:** Remote exam delivery, Online assessment, Open-book test, Multiple choice question (MCQ), Medical student

## Abstract

**Background:**

The use of remote online delivery of summative assessments has been underexplored in medical education. Due to the COVID-19 pandemic, all end of year applied knowledge multiple choice question (MCQ) tests at one UK medical school were switched from on campus to remote assessments.

**Methods:**

We conducted an online survey of student experience with remote exam delivery and compared test performance in remote versus invigilated campus-based forms of similar assessments for Year 4 and 5 students across two academic years.

**Results:**

Very few students experienced technical or practical problems in completing their exam remotely. Test anxiety was reduced for some students but increased for others. The majority of students preferred the traditional setting of invigilated exams in a computer lab, feeling this ensured an even playing field for all candidates. Mean score was higher for Year 4 students in the remotely-delivered versus campus-based form of the same exam (76.53% [SD 6.57] vs. 72.81% [6.64]; *t*_438.38_ = 5.94, *p* = 0.001; *d* = 0.56), whereas candidate performance was equivalent across both forms for Year 5 students.

**Conclusions:**

Remote online MCQ exam delivery is an effective and generally acceptable approach to summative assessment, and could be used again in future without detriment to students if onsite delivery is not possible.

## Background

Applied knowledge tests form an important component of assessment in undergraduate medical education. While the use of online technology for multiple choice question (MCQ) exam delivery is well-established [1, 2], most medical schools continue to run summative assessments on campus under invigilated conditions. This is particularly the case for higher stakes assessments such as final exams where the need to ensure test security is a major consideration [1].

The COVID-19 pandemic has forced universities to reassess their strategies for assessment as well as teaching, with most considering adopting some form of remote exam delivery as a means of future proofing against further periods of disruption. Remote exams have traditionally been open, allowing candidates free access to check information using resources during the assessment [1–3]. The relative merits of open versus closed approaches to assessment have been underexplored in medical education, with a recent systematic review [3] identifying few contemporary studies. To our knowledge, there has been little research evaluating online exam platform capabilities useful to the remote delivery of summative assessments, such as the ability to present questions in a random order to individual candidates [4].

However, legitimate concerns have been raised about potential inequalities in student experience in relation to remote online assessment [2, 5]. Not all students have access to up-to-date devices, reliable Wi-Fi, or a suitable quiet space for taking a remote exam [4, 5]. Such factors are likely to have a disproportionate impact on students from less-advantaged backgrounds and so have the potential to contribute to differential attainment.

Further evaluation of remote online exams in this context is clearly needed. In Edinburgh, the COVID-19 pandemic resulted in all end of year applied knowledge tests being run remotely for the first time. This provided an opportunity to compare the effects of remote versus invigilated computer lab delivery of similar assessments on students’ exam experience and academic performance.

## Methods

### Context

The Edinburgh MBChB is a 6 year programme with an integrated curriculum. Students complete a research-focused BMedSci degree in Year 3, with Year 4 being the first predominantly clinical year. Around half of students are Scottish/EU funded, with approximately 30% coming from the rest of the UK, and 20% being non-EU international students. 1n 2016, 8.3% of UK entrants to the programme fulfilled widening participation criteria.

Applied knowledge tests are used throughout the programme. Questions are based on clinical vignettes which candidates have to interpret and then determine the correct answer - such as the most likely diagnosis, next investigation or most appropriate management. The complexity of vignettes increases progressively through the programme. We use a mixture of best-of-five MCQs and very short answer questions. The latter comprise around 10% of exam content and are designed to be automatically marked by computer with a check by two faculty members to ensure all correct answers are identified.

All summative assessments are preceded by formative progress tests. Knowledge assessments are delivered via a commercial online platform (Practique®, Fry IT, UK) and are normally held in a university computer lab under invigilated conditions. Each exam is constructed to a predefined two dimensional blueprint based on clinical specialties and skills. Items are standard set by expert group using a modified Angoff approach, with previous performance data including Rasch modelling being taken into consideration for established items. Exams are time-limited based on a nominal 90 s per item (with up to 25% additional time for candidates with a learning adjustment).

All students had previous experience of using the exam system remotely for open-book formative assessments completed without strict time limits. The exam software is not dependent on continuous Wi-Fi connectivity, with the exam being downloaded onto devices at the start and candidate responses synching to a cloud-based server when Wi-Fi is available.

#### Impact of COVID-19 on assessment

Assessments were completed as normal until the end of February 2020. At the onset of lockdown in March in the UK many of our students returned to their family homes. We decided to continue with all remaining applied knowledge tests as non-invigilated remote exams using the time limits originally planned in order for progression decisions to be made. Students were advised to try and complete the exam as normal but that they could make strategic use of resources if necessary. Candidates sat their exam synchronously. We had students in time zones from + 7 to − 8 h BST, so chose a start time of 2 pm BST for each exam. An academic conduct statement and own work declaration had to be read and agreed on the initial screen in order to enter the exam. Items were presented in a random order to each candidate to reduce opportunities for collaboration.

This study includes Year 4 and Year 5 students. All Year 4 students completed the applied knowledge test (160 items) at the end of the second semester, whereas in Year 5 the cohort is split in half, alternating placements and related assessments (total of 170 items) between semesters. All assessments had satisfactory internal consistency (Crohnbach’s alpha around 0.85).

### Survey

We conducted an anonymous online survey to explore student experience of remote delivery in terms of exam accessibility and setting, test anxiety and preference. We chose to focus the survey on Year 5 students given their greater experience with online exams, and the fact they formed a natural crossover group in terms of remote versus invigilated on campus completion of the two components of their exam.

Following release of exam results (around 2 weeks after sitting the assessment), we sent all Year 5 students an invite to complete the survey. Participation was voluntary. Survey items are summarised in Table 1. We determined the frequency of categorical responses and identified emergent themes in free text comments.

**Table 1:** Remote exam experience survey items

***Setting and accessibility:***	
Did you have easy access to a suitable quiet space to sit the remote exam? (Yes/No)	
Did you have easy access to a suitable device for the remote exam? (Yes/No)	
Did you use a shared or borrowed device? (Yes/No)	
How was the functionality of the exam software on your device compared to the pcs in the computer lab? (much better/better/ the same/worse/much worse)	
Did you experience any significant Wi-Fi issues during the exam? (Yes/No)	
Any comments on setting/accessibility? (Free text)	
***Test anxiety:***	
Were you more anxious beforehand about the prospect of sitting the exam remotely compared to in the computer lab? (Yes/No)	
How anxious did you actually feel during the remote exam compared to the Semester 1 exam in the computer lab? (much more/more/the same/ less/much less)	
Can you explain the reasons for your responses? (Free text)	
***Preference:***	
Which exam experience did you prefer? (computer lab/no preference/remote)	
Can you explain the reason(s) for your preference? (Free text)	

### Performance

We compared candidate performance between remotely delivered assessments and the similar invigilated campus-based form of the exam from the previous academic year. Exam results are expressed using our institutional common marking scheme where raw scores are converted to a percentage and scaled, with the cut score equating to 60%.

Differences between groups were assessed using Welch’s modified two-sample t-test. The results were sufficiently normally distributed to make this a feasible choice. The analysis was adequately powered to detect a medium effect size (Cohen’s *d* = 0.3, with α = 0.05) [6]. All statistical analyses were performed in R [7].

### Ethics approval

The study was approved by the institutional medical education research ethics committee and informed consent obtained from all survey participants.

## Results

All candidates (*n* = 447) were able to complete their remote exam as planned, with 67 students (15.0%) accessing their assessment from outside the UK. Only 10 students (2.2%) contacted administrative staff about technical issues during their exam - all of which were minor and able to be resolved in real time - and these students were given extra time to compensate (maximum required was 10 min). One student made prior arrangements to sit the exam on campus using their own device in view of known issues with their home Wi-Fi. In terms of device choice, almost all students used a laptop computer.

### Student experience

The survey was completed by 119 Year 5 students (56.4% response rate).

#### Setting and accessibility

**Fig. 1:**
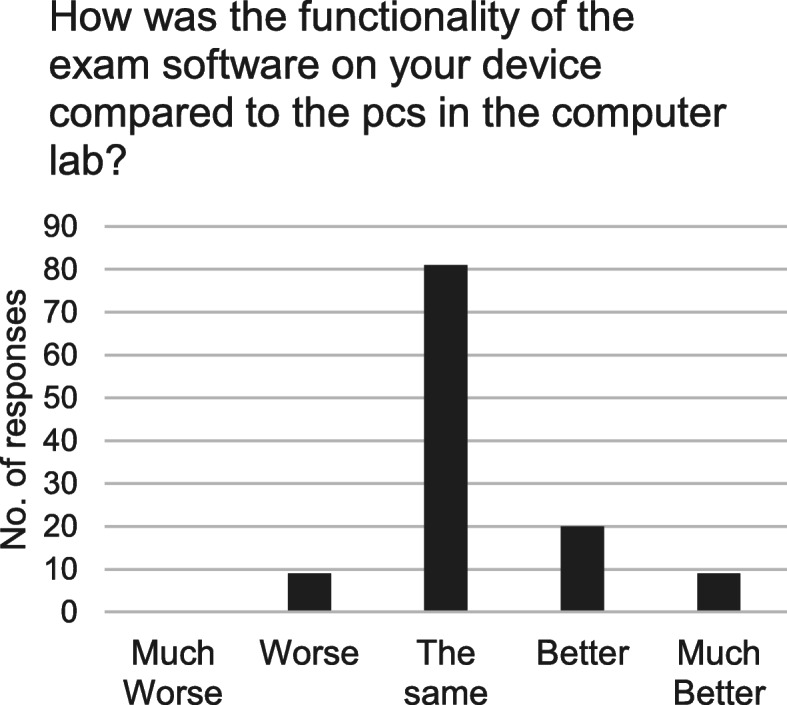
Remote functionality of the exam software (total responses *n* = 119)

All students indicated they had access to a suitable laptop or tablet for the remote exam, with this being a shared or borrowed device for 15 students (12.6%). Twenty-two students (18.5%) identified difficulties finding a quiet space for sitting the exam. Free text comments suggested the main issue was ambient noise from family, flatmates or neighbours during the exam. 10 students (8.4%) noted Wi-Fi issues during their exam. It is unclear whether these are the same students that contacted an administrator during the exam. Functionality of exam software on students’ own devices appeared to be at least as good as on the desktop pcs in the computer lab (Fig. 1). However, a few students felt images were more difficult to interpret with a smaller screen size.

#### Test anxiety

**Fig. 2:**
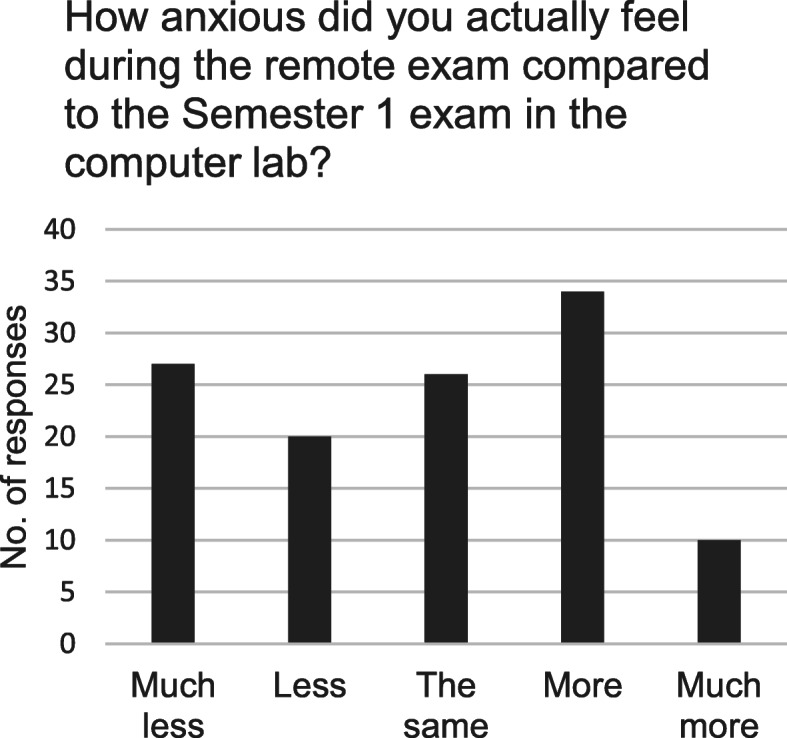
Candidate anxiety during the remote exam (total responses *n* = 117)

Opinion was split regarding anxiety in anticipation of sitting the remote exam with 61 students (51.3%) reporting feeling more anxious beforehand. There was a similar spread in anxiety levels experienced during the exam (Fig. 2). A number of students felt more relaxed sitting the exam remotely while a similar number felt more anxious, mainly due to concerns about technical issues like Wi-Fi connectivity (Table 2).

**Table 2:** Illustrative student comments relating to test anxiety

“Having to sit an exam at home is difficult, as you haven’t built associations between the exam and the environment in the same way you have with the computer lab. It can be more difficult to concentrate for the full 2 h. Furthermore, there’s stress associated with whether or not you’ll be able to connect etc.”	
“Computer lab feels familiar and is a professional setting which makes me feel more confident and relaxed. More standardised and fair for all students taking the exam.”	
“Being able to sit the exam at home really helped prevent the last-minute anxiety and nerves that I usually get with exams. I think this is the first exam I have ever had where I felt more at ease. The absence of the 30 min waiting time in a lecture theatre prior to the exam was ideal - less time to ruminate and worry. The waiting time is normally my biggest anxiety.”	
“Felt much more relaxed and comfortable sitting at home and was able to get up to walk about, have quicker toilet breaks, have drinks and snacks during, talk through things out loud to myself. Was able to use techniques to help anxiety and to boost concentration that I wouldn’t have been able to use in a group setting in the lab.”	

#### Preference

**Fig. 3:**
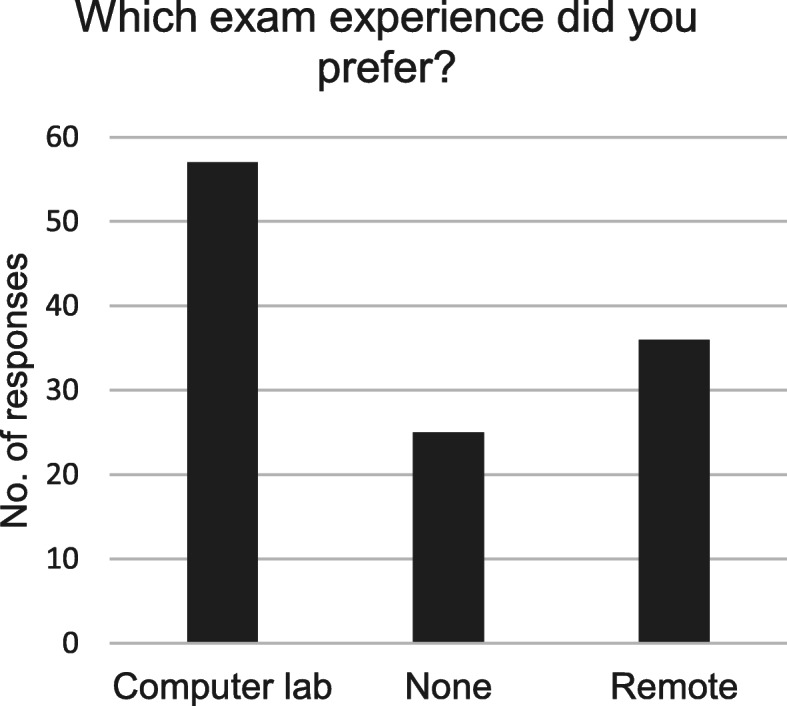
Candidate preference for exam setting (total responses *n* = 118)

Overall, the majority of students preferred sitting summative MCQ exams under invigilated conditions in the computer lab (Fig. 3), citing familiarity, positive associations, and standardisation as the main reasons for this (Table 2). Additionally, some students clearly felt uncomfortable with the blurring of boundaries caused by sitting a higher stakes exam in their own personal space. The issue of perceived fairness was mentioned frequently in free text comments, both in relation to accessibility and to the fact that those who had access to help from near-peers or medical friends and family during the exam might use this to gain an unfair advantage.

### Exam performance

Results are summarised in Table 3. Year 4 students performed better in the remotely-delivered compared to campus-based form of the same exam, with a medium effect size (*d* = 0.56) [6], whereas candidate performance was equivalent for Year 5 students. Variance across the two forms of each exam was similar in both year groups. Scores in both Year 5 semester exams were highly correlated (*r* = 0.54 [CI 0.44–0.63], *p* < 0.0001]), similar to the previous academic year when both were invigilated exams (*r* = 0.58 [CI 0.49–0.66], *p* < 0.0001).

**Table 3:** Comparison of remote vs. computer lab delivery of online applied knowledge tests

Exam	Remote (2020)			Computer lab (2019)			
	Candidates (n)	Mean score (%)	SD	Candidates (n)	Mean score (%)	SD	Test statistic for difference
Year 4	236	76.53	6.57	211	72.81	6.64	*t*_438.38_ = 5.94, *p* = 0.001^*^
Year 5 Paper A	107	77.25	9.43	119	76.02	8.41	*t*_213.66_ = 1.03, *p* = 0.30
Year 5 Paper B	104	73.68	8.75	121	72.21	7.91	*t*_209.71_ = 1.31, *p* = 0.20

Note. ^*^signifies statistically significant result

## Discussion

Student experience of remote online summative MCQ exam delivery during the COVID-19 pandemic was generally positive, with few students experiencing technical or practical problems. We did not find any evidence of negative effects on candidate performance.

### Test anxiety

Test anxiety was an important consideration given the context of these exams taking place during a global pandemic. Undoubtedly lockdown had unpredictable effects on individual students’ learning, revision and mental wellbeing as they adapted to a new normal [8], and this may have contributed to the heightened anxiety experienced by some students. It is interesting that the higher stakes nature of sitting a summative assessment seems to have resulted in many of our students worrying about access issues, particularly initial log in to the exam, despite their familiarity with using the system remotely. The literature suggests less test anxiety with remote delivery [1, 3], and we confirmed that this remained the case for a number of students where individual personality traits, learning styles or relief from negative associations with exam hall settings seem to have outweighed potential anxiety generated by the lockdown situation.

Test anxiety has been usefully conceptualised using distractional theories, in particular attentional control theory [9]. Addressing a task requires working memory to focus attention on several pieces of relevant information while inhibiting irrelevant information. According to attentional control theory, anxiety disrupts the balance between two competing attentional systems, with the one influenced by salient stimuli becoming stronger at the expense of the goal-directed system [9]. As working memory has a limited capacity, the addition of stress therefore reduces a candidate’s ability to use relevant information during a test resulting in underperformance. However, despite perceptions of increased or decreased test anxiety in our study, there was no obvious indication that any candidates actually did better or worse than predicted from previous performance.

### Test security

Although concerns have been expressed about increased opportunities for cheating during remote assessments, there is lack of objective evidence that this is more widespread than in invigilated campus-based exams [10]. We did not use remote proctoring but, in accordance with the argument put forward by Fuller et al. [5], invested trust in our students to behave professionally during remote exams, reminding them of appropriate conduct immediately before the start of their test. The ability to present items in a random order to individual candidates and the time-limited nature of the tests were felt to be adequate countermeasures against attempted collaboration. We did not take any steps to formally verify candidate identity for these assessments, e.g. by webcam, due to cohort sizes, simply sending the specific exam entry pass code to students’ university email accounts immediately before the exam started. As previously discussed, there were no candidates whose actual performance was unexpectedly greater than predicted from their previous performance in formative progress tests.

### Test performance

The remote online assessments were open-book by default and arguably the ability to quickly access and appraise relevant information to support clinical decision making is now a vital part of modern medical practice, underpinning professional values and patient safety. As such, testing candidates’ ability to do this by allowing access to resources during time-limited exams enhances their validity. This approach is already established in must-pass examinations such as the UK Prescribing Safety Assessment [11], where candidates have access to an online reference drug formulary.

We found that remote delivery aided candidate performance in the Year 4 exam, but not in Year 5. This was most likely due to Year 4 questions being less complex and covering more basic diagnosis, investigation and management of core and common medical conditions and therefore easier for candidates to check information to help them reach the correct answers. Our results contrast with the findings of Sam et al. who recently published a brief report of their experiences using an almost identical approach to exam delivery during the pandemic, but found no change in performance in any year group [12].

### Item leakage

Finally, the resource implications of running summative exams remotely have to be acknowledged. All items used have effectively been released into the public domain precluding their inclusion in future summative assessments. This approach therefore has significant costs in terms of replenishing exam item banks.

### Limitations

This was a naturalistic inquiry rather than pre-designed cohort study, comparing results from similar, but non-identical, assessments and across different time periods and therefore caution has to be taken in interpreting the results.

The generalisability of our findings is limited by this being a single institution study. In particular, we have a relatively low proportion of widening participation students who may be more likely to be disadvantaged by the remote approach.

While our results are encouraging it is difficult to clearly separate any effects arising specifically from lockdown.

## Conclusions

In the context of the COVID-19 pandemic, remote delivery of summative online applied knowledge tests was an effective and generally acceptable option for all our students with no evidence of detriment to candidate performance. As such we would be happy to use this approach again in future if necessary.

## Data Availability

The datasets used and/or analysed during the current study are available from the corresponding author on reasonable request.

## Abbreviation

MCQ: Multiple choice question
